# HDV RNA Levels and Progression of Hepatitis Delta Infection: A 14 Year Follow Up Experience in Italy

**DOI:** 10.3390/cells12101413

**Published:** 2023-05-17

**Authors:** Alessandra Mangia, Maria Maddalena Squillante, Filippo Fraticelli, Maria Chiara Cavorsi, Giulia Paroni, Lucia Zaffarano, Annarita Valeria Piazzolla

**Affiliations:** 1 Liver Unit, IRCCS Fondazione “Casa Sollievo della Sofferenza”, 71013 San Giovanni Rotondo, Italy; 2 Blood Bank, IRCCS Fondazione “Casa Sollievo della Sofferenza”, 71013 San Giovanni Rotondo, Italy

**Keywords:** quantitative HDV RNA, cirrhosis, HBV DNA, quantitative HBsAg

## Abstract

Background: Identification of outcome predictors is one of the unmet needs in chronic HDV infection. Until recently, no reliable quantitative assays for HDV RNA were available. Aims: To evaluate the impact of baseline viremia on natural history of HDV infection in a cohort of patients whose serum samples were stored at their first visit 15 years ago. Methods: Quantitative HBsAg, HBeAg, HBeAb, HBV DNA, HDV RNA, genotypes, and liver disease severity were assessed at baseline. Patients who were no longer on active follow-up were recalled and re-evaluated in August 2022. Results: The majority of patients were male (64.9%); the median age was 50.1 years; and all patients were Italian, with only three born in Romania. All were HBeAg negative with HBV genotype D infection. Patients were subdivided three groups: 23 were in active follow-up (Group 1), 21 were recalled due to no longer being in follow-up (Group 2), and 11 died (Group 3). Liver cirrhosis was diagnosed in 28 subjects at the first visit; 39.3% of diagnosed patients were in Group 3, 32.1% were in Group 1 and 28.6% were in Group 2 (*p* = 0.001). Baseline HBV DNA IU/mL Log10 were 1.6 (1.0–5.9) in Group 1, 1.3 (1.0–4.5) in Group 2, and 4.1 (1.5–4.5) in Group 3; median baseline HDV RNA Log10 levels were 4.1 (0.7–6.7) in Group 1, 3.2 (0.7–6.2) in Group 2, and 5.2 (0.7–6.7) in Group 3, resulting significantly higher rates among patients in Group 3 compared to the other groups (*p* = 0.038). Eighteen patients in Group 2, as compared to 7 in Group 1, had undetectable HDV RNA at the follow-up evaluation (*p* = 0.001). Conclusions: HDV chronic infection is a heterogeneous disease. It may not only progress but also improve over time in patients, who eventually become HDV RNA-undetectable. HDV RNA levels may help identify the subgroup of patients with less progressive liver disease.

## 1. Introduction

Hepatitis delta virus (HDV) is responsible for one of the most severe forms of chronic liver disease, with rapid progression to cirrhosis and hepatocellular carcinoma (HCC) [1]. HDV is a single-stranded RNA virus requiring the helper function of the HBV surface antigen (HBsAg) for hepatocyte infection [2]. Recent estimates suggest that more than 12 million subjects are globally delta antibody-positive (anti-HDV) [3,4]. In Italy, the infection declined since the widespread introduction of HBV vaccination after 1990 [5]. Although recent migratory waves from high-endemic areas are associated with increasing prevalence, domestic population infection peaked 25 years ago [6].

HDV chronic infection was traditionally associated with the most severe form of liver disease and clinical decompensation; however, evidence in support of a milder course exists [7,8]. In a study performed in our geographical area in 1996, a long-lasting disease with biochemical features of mild active or inactive liver disease was observed in 10% of 122 patients [7]. Almost one-third of them were treated with interferon alfa. Another retrospective study from Spain, published in 2021, reports a milder course of HDV chronic infection [8]. Of 56 patients with compensated liver disease, followed longitudinally for a mean duration of 5.6 years and treated in 2/3 of cases with nucleoside analogues, 25% reported HDV RNA decline ≥ 2 Log10 or HDV RNA undetectability. HDV RNA was assessed via in-house reverse-trascriptase-polymerase chain reaction (PCR), using the WHO international standard with lower limit of detection (LLOD) of 100 IU/mL and lower limit of quantitation (LLOQ) of 575 IU/mL. Patients with viral decline had a longer follow-up period but not a better clinical outcome. These results are in partial agreement with findings reported by Romeo et al. in 2014 in 193 patients with HDV chronic infection, 45% of whom had cirrhosis [9]. Using an in-house RT PCR, the authors demonstrated that the persistence of HDV RNA replication was associated with disease progression, including HCC development [9]. Overall, this evidence corroborates previous findings that HDV-related factors may be predictive of an adverse outcome in HDV chronic infection.

Previous studies on viremia levels were hampered due to a lack of standardization of HDV RNA quantitation in specialized centers [10]. Only recently was an international standard for HDV RNA measurement introduced that allowed the harmonization of commercial assays and HDV RNA extraction procedures, avoiding the limitations of comparison of quantitative results between different centers [11,12].

The unique availability of stored serum samples from a cohort of patients followed at our Unit since 2006 prompted us to explore the impact of HDV RNA levels on the natural history of HDV using a very sensitive quantitative commercial assay. We evaluated stored baseline samples together with last visit samples, in addition to liver disease severity and liver-related events, to investigate the predictive role of HDV RNA on disease progression.

## 2. Patients and Methods

Patients diagnosed at our Unit from 2006 to August 2022, who agreed to be followed, were enrolled if they had HBsAg positive, anti-HDV IgG positive, histological or clinical evidence of chronic liver disease, no HIV co-infection, and no evidence of alcohol abuse or other causes of chronic liver diseases. Diagnosis of HDV infection was confirmed via quantitative HDV RNA assessment in the stored blood sample at the time of diagnosis and at the last follow-up when anti-HDV results were positive.

Anti-HDV, anti-HCV, and anti-HIV were part of the clinical workup of our patients at the first visit, as were HBsAg, HbeAg, and HBeAb. HBV DNA was quantitatively evaluated in the same baseline stored samples used for delta retrospective evaluation, as well as quantitative HBsAg and HBV and HDV genotype. Patients with HCVAb serum samples were also tested for quantitative HCV RNA.

Baseline clinical and laboratory characteristics were collected from patients’ medical records.

Liver cirrhosis was diagnosed via liver biopsy, and histology was assessed using the Ishak score. In patients without available histology, diagnosis of cirrhosis was based on evidence of portal hypertension via ultrasound and endoscopic evidence of esophageal varices or Platelet count lower than 120.000 mm^3^.

Liver stiffness measurement through Fibroscan (Echosense, Paris, France) was available from 2007 onward. The study was carried out in accordance with the principles of the Helsinki declaration and with local and national laws. Approval was obtained from the Ethical Committee (NCT01401400; 2011).

### 2.1. Methods

Baseline and most recent serum HDV RNA levels were evaluated using the RoboGene HDV RNA quantification kit 2.0 (CFX96, BioRad Laboratories) (LLOD of 6 IU/mL) in the stored samples. Anti-HDV antibodies were performed using a commercial EIA assay (Dia.Pro. Diagnostic Bioprobes Srl., Sesto San Giovanni, Italy).

Serum HBV DNA levels were determined via a commercial quantitative PCR assay (Amplicor HBV Monitor 2.0 assay, RocheDiagnostic Systems Inc., Mannheim, Germany) with a LOD of 10 IU/mL and a lower limit of quantification of 20 IU/mL.

HCV RNA was evaluated via Abbott RT-PCR assay (Abbott Laboratories, North Chicago, IL, USA) with a LOD 12 IU/mL.

Quantification of serum HBsAg was performed via HBsAg (Architect, HBsAg assay, dynamic range 0.05–250.0 IU/mL, WHO standard; Abbott Laboratories, Chicago, IL, USA), after appropriate dilution, when >250 IU/mL.

HBV genotype was determined via Innogenetics INNO-LiPA HBV genotyping (Fujirebio Europe N.V., Zwijnaarde, Belgium). HDV genotyping was performed via direct sequencing of delta antigen region, as reported in [13].

### 2.2. Specific Treatments and Response Definition

As HDV-specific treatment, a subgroup of patients received either recombinant Interferon three times per week or PegInterferon once per week for 48 weeks, extended to a second course in case of biochemical response. Virological response was defined as undetectable HDV RNA in any stored serum sample post-treatment. A biochemical response was defined as normal ALT and AST levels during follow-up in patients with previously documented raised ALT and AST levels.

### 2.3. Liver Disease Assessment and Progression

Liver cirrhosis was diagnosed based on liver histology, liver stiffness >12.5 KPa, or clinical diagnosis. Progression of disease was defined as a composite endpoint including the following features: (1) evidence of liver cirrhosis complications, de novo hepatocellular carcinoma (HCC), or liver transplant (OLT); (2) evidence of worsened histological features; and (3) 20% increase in transient elastrometry results for patients diagnosed after 2007. Development of a dismal event, such as hepatic decompensation, HCC, OLT, or death, was the established endpoint.

### 2.4. Statistical Analysis

Baseline characteristics and measures of clinical and demographic characteristics are reported as median and interquartile range (IQR) values for continuous variables and frequencies for categorical variables. The primary analysis was related to the composite outcomes. One-way analysis of variance was used in the comparison of continuous data in different subgroups. The simple chi-squared statistic or Fisher’s exact test were applied for the analysis of frequencies for categorical data.

Parameters with a *p* value < 0.005 in the univariate analysis were included in multivariable analysis. *p* values < 0.05 were considered statistically significant. All analyses were performed by using SPSS version 25 (IBM Corp., Armok, NY, USA)

## 3. Results

### 3.1. Baseline Demographic and Virological Data

A total of 57 patients (20 females and 37 males) with a median age at baseline of 50.1 (21.0–70.0) represented the baseline study population. Two patients with active HCV infection were excluded. The median follow-up length after our clinic’s first visit was 14.5 (1–16.3) years. All but three patients were Italian descendants, with the exceptions being born in Romania. The route of HDV acquisition was unknown in 51.8% of cases. HDV infection was associated with intravenous drug use in 21.4% of cases, parenteral transmission in 7.2% of cases, and vertical transmission in 10.7% of cases. Intrafamilial transmission was ascertained in 8.9% of cases.

**Figure 1:**
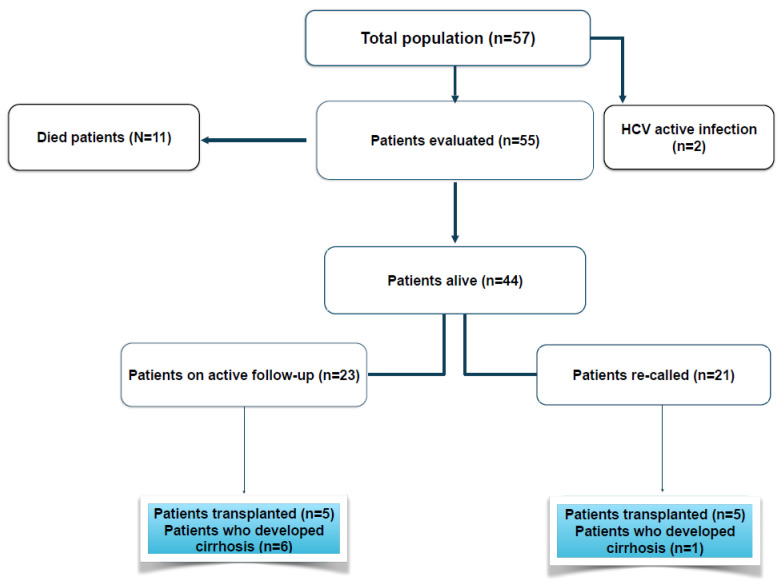
Flow chart of patients included in original cohort and final outcomes.

All patients tested HBsAg-positive at baseline, and all were HbeAg-negative. Overall, eight patients (14.0%) tested anti-HCV-positive, while all but two patients were successfully treated for HCV; the outliers were excluded. At baseline, 12 patients had HBV DNA below LOD (21.4%); of these patients, 5 were taking NAs, while 4 had been treated with IFN for 1 or 2 years in the past, experiencing a biochemical response as a result. In total, 52 (94.6%) had levels of HDV RNA higher than the assay threshold. The remaining three patient had HDV RNA at the assay threshold; none of them had cirrhosis. All the patients had HBV genotype D and HDV genotype 1. Of the 57 patients, 2 patients were excluded as previously reported, 11 have since died, and 44 are still alive (Figure 1).

Patients were subdivided into three groups, including patients who died of liver-related reasons (19.3%) (Group 3). Of the remaining patients, 23 (52.7%) were on active follow-up (Group 1), while 21 (47.7%) were not and were recalled for assessment (Group 2). Demographic features at baseline by group are reported in Table 1.

### 3.2. Clinical Presentation and Outcome after the First Visit

Overall, 28 patients (50.9%) had evidence of cirrhosis at baseline (Table 1), 24 had esophageal varices, and 4 had ascites. With the exclusion of patients who died, no variceal bleeding was reported. Of 28, 11 patients with cirrhosis were in Group 3 (39.3%), 9 were in Group 1 (32.1%), and 8 (28.6%) were in Group 2 (*p* = 0.001). All patients in Group 3 and a single patient in Group 1 had decompensated cirrhosis.

Of 11 deaths (20%), 7 were due to liver failure (in 3 cases, co-morbidities, such as sepsis, breast cancer, and severe diabetes, were present), 1 was due to variceal bleeding, and 3 were due to an advanced HCC at diagnosis. All dead patients had evidence of cirrhosis at the first diagnosis.

During the follow-up, 12 patients (21.8%) underwent OLT: for 8 patients, OLT treatment was required due to HCC, while for the remaining 3 patients, liver failure was the cause. No differences were observed in the proportion of transplanted patients as rates of 21.7%, 21.1%, and 27.3%, respectively, were observed across each group (*p* = 0.89).

Seven patients developed cirrhosis or HCC during the follow-up: six patients were in Group 1, while one was in Group 2 (*p* = 0.03); one patient underwent OLT in response to this diagnosis. Of the remaining patients, one individual in Group 1 and two in Group 2 showed improvement in TE results.

### 3.3. Biochemical, Virological Outcome in Patients Alive (Groups 1 and 2)

Baseline characteristics of patients by group are reported in Table 1. In 15 of 23 (65.2%) patients on active follow-up, ALT and AST levels decreased or normalized over time with median levels of 106.4 U/L and 104.6 U/L vs. 85.6 U/L and 91.2 U/L, respectively, at the end of follow-up. The corresponding features for patients included in Group 2 were as follows: nine of twenty-one (36.8%) experienced ALT and AST decline from 101.0 U/L and 88.8 U/L to 89.7 U/L and 65.9 U/L. Differences between baseline ALT and AST levels across groups were not significant (*p* = 0.72 and *p* = 0.07, respectively). At the last follow-up visit, ALT and AST decline was not statistically different for patients in Groups 1 and 2 (*p* = 0.15 and *p* = 0.37, respectively).

In Group 1, the median HBsAg IU/mL Log10 at baseline was 3.3 (0.6–4.4). It declined to 3.0 (0.6–3.8) in all patients with detectable HBsAg levels at the last visit. All patients with undetectable HBsAg were on intravenous IgG treatment after OLT (100%). In 12 of 22 patients in Group 2, HBsAg remained detectable. Median HBsAg IU/mL Log were 3.4 (0.6–4.3) at baseline and 0.9 at the last follow-up visit. Of 10 patients with undetectable HBsAg, only 4 (40.0%%) were on intravenous IgG treatment. With the exclusion of patients on intravenous IgG, no patients in Group 1 experienced HBsAg clearance, compared to six patients in Group 2.

Of 55 patients, 7 (1 in Group 1, 5 in Group 2, and 1 in Group 3) had baseline undetectable HBV DNA (*p* = 0.16). All patients took tenofovir disoproxyl fumarate or entecavir. Median baseline HBV-DNA IU/mL Log10 values were 1.6 (1.0–5.9) in Group 1, 1.3 (1.0–4.5) in Group 2, and 4.1 (1.5–4.5) in group 3 (*p* = 0.001). This difference was due to the higher HBV DNA levels in Group 3 (Table 1), as Log10 levels in Group 1 did not differ from those of Group 2 patients (*p* = 0.81). It should be noted that patients in Group 3 were exclusively treated with lamivudine or adefovir. At the last visit, HBV DNA was below LLOQ in 10 patients in Group 1, 15 patients in Group 2, and 2 patients in Group 3 (*p* = 0.021). All patients, except one individual who received PegIFN, were treated with NAs. When patients who died were excluded, no difference was observed in the number of patients with detectable HBV DNA in Groups 1 and 2 (*p* = 0.13).

**Figure 2:**
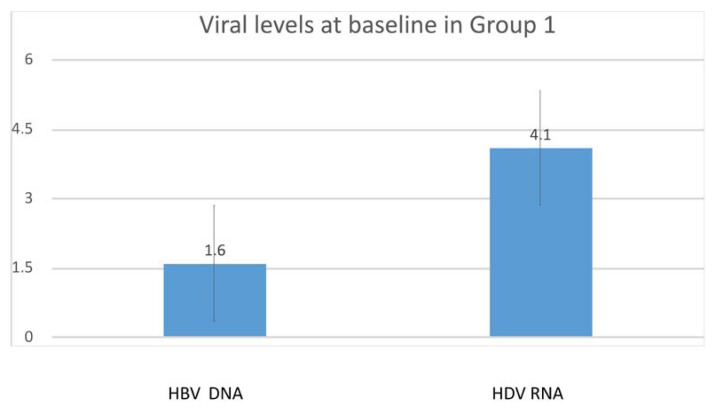

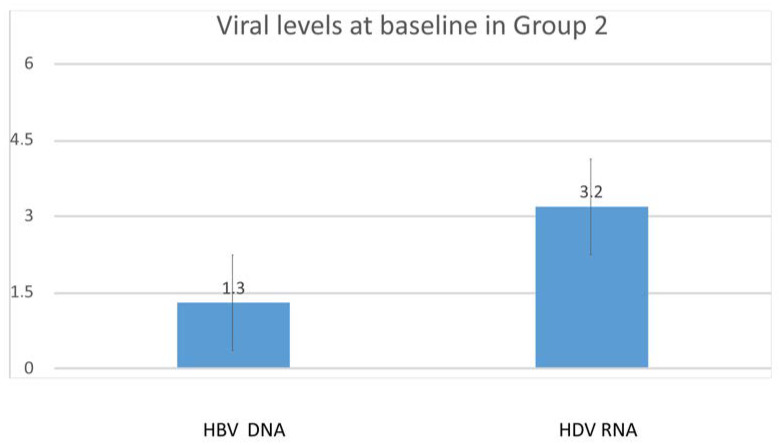
HBV DNA and HDV RNA levels at baseline by group of patients still alive at last follow-up visit.

All patients had anti-HDV antibodies higher than the assay threshold at baseline and the last follow-up visit. Baseline median HDV RNA IU/mL Log10 results were 4.1 IU/mL Log10 (0.7–6.7) in Group 1, 3.2 IU/mL Log10 (0.7–6.2) in Group 2 (Figure 2) (*p*= 0.11 for comparison between Group 1 and 2), and 5.2 IU/mL Log10 (0.7–6.7) in Group 3 (*p* = 0.038 for comparison of Group 1, 2 and 3). At baseline, one patient in Group 1, two in Group 2, and one in Group 3 tested HDV RNA-undetectable (*p* = 0.76). At the last visit, HDV RNA levels declined to 2.8 (0.7–6.7) in Group 1 and 1.2 (0.7–6.2) in Group 2 (*p* = 0.004). Seven patients were HDV RNA-negative in Group 1, compared to eighteen in Group 2 (*p* = 0.001). Of these patients, 4 and 5 were transplanted in Groups 1 and 2, respectively; after their exclusion, 3 patients in Group 1 were HDV RNA undetectable, compared to 13 in Group 2 (*p* = 0.001).

Overall, 35 non-transplanted patients were still alive at the last visit. Of them, 16 were HDV RNA-undetectable. Only one patient from group 1 had evidence of cirrhosis, while only eight had received a course of IFN. The virological profile of patients by outcome is reported in Table 2.

### 3.4. Biochemical and Virological Characteristics of Patients Who Died

The median age of 11 patients who died was not significantly higher than in Groups 1 and 2 (52.0 vs. 53.0 and 44.1, respectively) (*p* = 0.70). The first visit of these patients was performed 15.6 years before the present analysis, while the interval between baseline and last follow-up visit for Groups 1 and 2 were 11.1 and 15.1, respectively (*p* = 0.07). In Group 3, the baseline median ALT level was 91.4 U/L (*p* = 0.38 for comparison with Group 1 and 2), while the median AST levels was 101.2 U/L (*p* = 0.10 for comparison with Group 1 and 2). The median baseline HBsAg IU/mL Log10 level was 3.5 (2.9–4.6) (*p* = 0.28 for comparison with Group 1 and 2). Probably due to a weaker viral control, HBV DNA levels were significantly higher than in Groups 1 or 2.

As reported, baseline levels of HDV RNA were significantly higher than in Groups 1 or 2 (Table 1). No differences were observed between HBV DNA levels in patients in Groups 1 and 2 (Table 1).

### 3.5. Baseline Predictors of Favourable Outcome

In the entire cohort, baseline factors associated with unfavorable outcome via univariate analysis included age and gender, the duration of follow-up, the assigned group, PLTs, cirrhosis, HBV DNA IU/mL Log10 results at baseline, HDV RNA IU/mL Log10 results at baseline, IFN treatment, the baseline Log10 HDV RNA IU/mL (*p* < 0.001), baseline Log10 HBsAg IU/mL (*p* = 0.002), the assigned group (*p* = 0.003), PLTs (*p* < 0.001), and cirrhosis (*p* = 0.001). The use of NAs was inversely associated with the unfavorable outcome and was considered as an expression of a more severe disease that did not suit the use of IFN. Among 13 patients with HDV RNA IU/mL <1100, only 1 developed cirrhosis.

Using the multivariate model, the only independent predictor of unfavorable outcome was higher HDV RNA IU/mL Log10 (*p* value = 0.018, OR 0.39, 95% CI 0.18–0.84).

## 4. Discussion

Our data suggest that HBV/HDV infection might not always be rapidly progressive. In our HBeAg negative patients, the lower the baseline HDV RNA levels, the higher the likelihood of not developing cirrhosis. In particular, a threshold of 1000 IU/mL HDV RNA might be identified as suggestive of a lower risk of progression to cirrhosis, HCC, or OLT.

Different clinical manifestations of HDV chronic carriers, ranging from a stable mild liver disease escaping close physician monitoring (Group 2) to a severe and rapidly progressive disease requiring strict HCC monitoring or OLT indication (Group 1 and 3), were observed in our series and drove our risk stratification. Of 35 non-transplanted patients still alive at the last visit, which occurred 15 years after the first, 16 (45.7%) currently show undetectable HDV RNA; this rate is significantly higher in Group 2 than in 1. HDV RNA undetectability at the last visit was associated with HBsAg- and HBV DNA-negative results in 92.9% of cases. Higher baseline HBV DNA levels—despite a week viral suppression—in addition to higher baseline HDV viremia correlate with an unfavorable outcome in Group 1. Our data confirm the known relationship between HDV and HBV in HBeAg negative/antiHBe carriers, showing a less aggressive course of disease in patients with lower baseline HDV RNA levels despite ongoing HBV replication [14,15,16].

Overall, 22.4% of non-transplanted, living non cirrhotic patients with undetectable HDV RNA never received treatment. In keeping with other studies [17], we suggest that intrinsic characteristics of HDV/HBV interaction in our geographical area might influence the course of the disease.

The paramount role of HDV RNA quantification in monitoring treatment was clearly remarked in chronic HDV infection [12]. However, until recently, the lack of reliable, standardized HDV RNA diagnostic assays hampered our understanding of the impact of viremia in the natural history of HDV-associated liver disease [12]. In our study, at variance with previous studies [7,8,9], a commercial standardized assay rather, than an in-house RT quantitative PCR, was used. The higher sensitivity and manual extraction increase the accuracy of our measurements and the meaning of the associations between baseline HDV RNA levels and clinical outcomes. Another study from Sweden recently investigated the role of viremia levels in 233 patients of different races and geographical origins, who had detectable HDV RNA and a mean follow-up of 6.5 years (range 0.6–33) [18]. At variance, our study focuses on patients from the same country and, in the vast majority of cases, the same geographical area of origin.

Until recently, Interferon was the only available treatment for hepatitis delta [19,20,21]. The impact of Interferon on the natural history of HDV chronic hepatitis is debated. Interferon is associated with low response rates, although a long-term follow-up study showed that high doses of interferon alpha-2a given for many years significantly improved long-term outcomes and survival rates for a small cohort of patients with chronic HDV infection [21]. In a recent meta-analysis, Peg-IFN was associated with one-third of patients achieving viral clearance and ALT normalization [22]. Moreover, the late relapse risk further reduces this treatment’s efficacy [23]. In our study, 27 patients (77.1%) of those yet alive had received a course of IFN, but only 8 (29.6%) showed undetectable HDV RNA levels and an absence of cirrhosis at the last visit. Almost one-third of patients were on NAs treatment at the first observation, with the rate increasing to 88% over time. All but one patient developed cirrhosis despite NAs, confirming the poor outcome of HDV infected patients treated with NAs [24].

Liver transplant is a valuable therapeutical option in HDV patients (25,26). Nine (20.4%) of our forty-four living patients were previously transplanted. As shown by Samuel et al., the administration of immunoglobulins against HBsAg reduces the risk of reinfection in these patients [25]. This outcome was the case for 6 of our transplanted patients. In keeping with previous reports [26,27], the use of potent NAs as tenofovir or entecavir adopted in three additional cases was also associated with absence of reinfection.

The high death rate shown in our study (20%) could be the consequence of the late diagnosis that, until recently, was associated with HDV-related disease. Indeed, cirrhosis was already diagnosed at the first observation in all patients who died. Moreover, their age was slightly higher than that of patients from the other groups, suggesting that the disease was acquired in the era of suboptimal HBV treatment.

In addition to the availability of the unique sample collection at the first observation at our clinic, the strengths of our work are the extended to cover the follow-up period and the homogeneous demographic characteristics of our study population. A common HBV genotype D and HDV genotype 1 infection, avoiding the risk of different outcomes influenced via different HDV genotypes [28], reinforces the relevance of our results, which is at variance with studies including migrants of different geographical origins [29]. Moreover, another advantage is the accurate, highly sensitive, and standardized method used to determine HDV RNA, which uses internal control samples quantitated in other laboratories. Finally, the possibility of recalling all patients no longer on active follow-up allowed us to provide a complete idea of the natural history of HDV-associated liver disease in real life.

The limitations of this work should also be acknowledged. These limitations include the retrospective nature and the limited sample size, although it should be considered that our series is numerically comparable with others previously published [8]. In addition, someone may argue that using only baseline and last visit changes in clinical parameters is not sufficient to prove that HDV RNA has a predictive value.

In conclusion, our study sheds light on the unmet need of overcoming lack of outcome predictors in HDV chronic infection. We demonstrated that HDV chronic disease might not always be rapidly progressive. A subgroup of patients with suppressed HBV DNA replication and low baseline HDV RNA levels may experience undetectable HDV RNA and absence of cirrhosis over time.

In newly diagnosed cases, assessing HDV viremia at baseline may help in therapeutic decision-making.

The advent of new and safe therapeutical approaches, and the possibility to use validated and standardized HDV diagnostic tests before and during treatment, will reduce the number of subjects lost during the follow-up period.

## Figures and Tables

**Table 1:** Baseline characteristics of patients by study group.

Characteristics	Patients on Active Follow Up (Group 1) (*n* = 23)	Patients Re-Called (Group 2) (*n* = 21)	Patients Died (Group 3) (*n* = 11)	*p* Value
Age, yrs, median, IQR	53.0 (21.0–69.0)	44.1 (25.3–70.0)	52.0 (40.1–59.2)	0.70
Male sex, *n* (%)	15 (65.2)	14 (66.6)	7 (63.6)	1.0
Prior drug abuse, *n* (%)	4 (17.4)	6 (28.5)	2 (18.2)	0.68
HBV DNA > 2000 IU/mL, *n* (%)	19 (82.6)	15 (71.4)	10 (90.9)	0.51
HBV DNA level log10 ^§^ IU/ml	1.6 (1.0–5.9)	1.3 (1.0–4.5)	4.1 (1.5–4.5)	0.001
HBsAg level log10 IU/ml	3.3 (0.6–4.4)	3.4 (0.6–4.3)	3.5 (2.9–4.6)	0.28
HDV RNA level log10 IU/ml	4.1 (0.7–6.7)	3.2 (0.7–6.2)	5.2 (0.7–6.7)	0.038
ALT U/L	91.2 (27.0–426.1)	57.2 (16.1–369)	91.4 (59.1–432.0)	0.38
Previous IFN treatment ^ *n*, (%)	12.0 (52.2)	12.0 (57.1)	4 (36.4)	0.11
NAs treatment *n*, (%)	15 (65.2)	7 (33.3) *	2 (18.2)	0.017
PLTs 109/L median (IQR)	142.2 (27.1–225.0)	143.1 (22.1–200.3)	108.0 (19.0–113.1)	0.09
Liver stiffness value kPa, median (IQR)	13.2 (10.53–16.1)	11.9 (6.3–20.1)	14.0 (12.6–26.0)	0.38
Liver cirrhosis at baseline, *n* (%)	9 (39.1)	8 (38.1)	11 (100.0)	0.004
Follow-up duration, yrs, median (IQR)	11.1 (8.1–15.3)	15.1 (4.1–15.8)	15.6 (11.0–16.3)	0.07

^§^ *p* = 0.001 for comparison between Groups 1 and 3; ^ 9 patients overall (1 in group 1 and 8 in Group 2) had not received either IFN or NAs; * *p* = 0.69 for comparison between Groups 1 and 2; *p* > 0.01 for comparison between Groups 2 and 3; *p* = 0.81 for comparison between Groups 1 and 2.

**Table 2:** Baseline predictors of unfavorable outcomes.

Characteristics	Cirrhosis/OLT/Death at Last Follow Up 30	No Cirrhosis/OLT/Death at Last Follow Up 25	*p* Value
Age, yrs, mean, SD	54.0 (25.3–69.0)	44.8 (20.0–70.0)	0.45
Male sex, *n* (%)	19.0 (63.3)	16.0 (64.0)	1.0
Prior drug abuse, *n* (%)	6 (20.0)	6 (24.0)	0.75
HBV DNA > 2000 IU/mL, *n* (%)	5 (16.7)	8 (32.0)	0.21
HBV DNA level log 10 U/ml	2.1 (1.0–4.8)	1.6 (1.0–5.9)	0.25
HBsAg level log10 IU/ml	3.54 (2.6–4.7)	3.0 (0–4.62)	0.047
HDV RNA level log 10 IU/ml	5.2 (2.3–6.6)	3.0 (0.7–5.92)	0.029
HDV RNA level log10 < 3, *n* (%)	6 (20.0)	12 (48.0)	0.043
ALT IU/ml	100.4 (30.1–432.0)	63.0 (16.1–426.1)	0.21
IFN treatment *n*, (%)	17 (56.7)	12 (48.0)	0.59
NAs treatment *n*, (%)	19 (63.3)	5 (20.0)	0.002
PLT count 109/L median (IQR)	108.5 (19.1–160.3)	167.1 (22.1–225.0)	0.028
Liver stiffness value kPa, median (IQR)	13.3 (8.0–28.1)	9.0 (4.7–21.0)	0.55
Liver cirrhosis at baseline, *n* (%)	25 (83.3)	3 (16.7)	0.0001
CPT classes, *n* (%)			0.82
A	18 (72.0)	1 (5.3)	
B	6 (24.0)	0	
C	1 (4.0)	0	
Group 1	11 (36.7)	12 (48.0)	0.003
Group 2	8 (26.7)	13 (52.0)	
Group 3	11 (36.7)	0	
Follow up duration, yrs, median (IQR)	15.2 (1–16.3)	14.5 (1–15.3)	0.10

## Data Availability

Data are available at https://zenodo.org/record/5728042#.Ycy6BWnsJPw (accessed on 12 June 2022).
